# Trajectory of Irisin as a Predictor of Kidney-Related Outcomes in Patients with Asymptomatic Heart Failure

**DOI:** 10.3390/biomedicines12081827

**Published:** 2024-08-12

**Authors:** Tetiana A. Berezina, Oleksandr O. Berezin, Uta C. Hoppe, Michael Lichtenauer, Alexander E. Berezin

**Affiliations:** 1 Department of Internal Medicine and Nephrology, VitaCenter, 69000 Zaporozhye, Ukraine; talexberezina@gmail.com; 2 Departament of Alter Psychiatrie, Luzerner Psychiatrie AG, 4915 St. Urban, Switzerland; 3 Department of Internal Medicine II, Division of Cardiology, Paracelsus Medical University, 5020 Salzburg, Austria; u.hoppe@salk.at (U.C.H.); m.lichtenauer@salk.at (M.L.)

**Keywords:** asymptomatic heart failure, heart failure with preserved ejection fraction, heart failure with mildly reduced ejection fraction, type 2 diabetes mellitus, kidney-related outcomes, irisin, serial measurements

## Abstract

The purpose of the study is to elucidate whether irisin is a promising predictive biomarker for kidney-related events in patients with T2DM and concomitant asymptomatic HF. We prospectively enrolled 146 T2DM patients who had either evidence of structural cardiac abnormality or elevated levels of N-terminal brain natriuretic pro-peptide (NT-proBNP) > 125 pmol/mL and followed them for 52 weeks. Structural cardiac abnormalities were used as the minimum from the following criteria: abnormal left ventricular (LV) global longitudinal strain (GLS) < −16%, LV hypertrophy, left atrial volume index > 34 mL/m^2^, abnormal ratio of early transmitral diastolic filling velocity/early mitral annular velocity ≥ 13 units. All the patients underwent echocardiographic and Doppler examinations by two blinded, highly experienced echocardiographers. NT-proBNP, irisin, TNF-alpha, and hs-CRP were quantified in the serum at baseline, at 26 weeks, and at the end of the study. The kidney-related outcomes consisted of an eGFR reduction by 40% from baseline, or end-stage kidney disease, or kidney replacement therapy. We found that levels of irisin at baseline < 4.15 ng/mL and/or its decrease > 20% from baseline in T2DM patients predicted kidney-related events better than baseline levels/dynamic NT-proBNP and the use of SGLT2 inhibitors. In conclusion, we established that a low baseline level of irisin and its 20% decrease correlated with newly kidney-related events in T2DM patients with asymptomatic HFpEF/HFmrEF.

## 1. Introduction

In recent decades, the global prevalence of type 2 diabetes mellitus (T2DM) has demonstrated a steady trend to increase due to the aging of the population, an elevated prevalence of metabolic risk factors, and genetic/epigenetic causes widely penetrating in different ethnicities and genders [1,2]. Individuals with T2DM are at a higher risk of developing a range of complications including diabetic cardiomyopathy, ischemia- and non-ischemia-induced heart failure (HF), chronic kidney disease (CKD), and coronary artery disease (CAD), which intervene in terms of the prognosis and quality of life [3]. Potential molecular mechanisms contributing to functional, morphological, and structural impairments, known as diabetes-induced cardiomyopathy, affect altered cardiac metabolism, and are the result of the following: glucose and lipid toxicity, mitochondrial DNA damage, insulin resistance and impaired cardiac insulin metabolic signaling, cardiac myocytes endoplasmic reticulum stress, mitochondrial stress and dysfunction due to oxidative stress, inflammation, neurohumoral activation and adipose tissue dysfunction, impaired vascular integrity, and endothelial dysfunction [4,5]. These pathophysiological factors link CV and metabolic conditions with myocardial cell death pathways, the over-expression of pro-inflammatory genes, the accumulation of extracellular matrix and fibrotic changes, and cardiac autonomic neuropathy, which are considered the main causes leading to adverse cardiac remodeling and consequently the development and progression of HF with a preserved (HFpEF) and reduced/mildly reduced (HFrEF/HFmrEF) ejection fraction [6,7].

CKD is a common comorbidity in T2DM patients that is increasing in prevalence and affects about 50% of T2DM patients globally [8]. It has been found that HF along with the duration of T2DM are the most important risk factors related to a reduction in the glomerular function rate (GFR) and contribute to kidney-related events [9,10]. On the contrary, worsening GFR is frequently associated with the development of HFpEF, which in non-dialysis CKD individuals is the second cause of death after CAD [11,12]. Meanwhile, besides the serial assessment of creatinine levels, which are biomarkers of kidney damage, no other approach to the detection of CKD has been suggested, while numerous promising biomarkers are widely considered [13]. There is limited evidence of circulating biomarkers regarding the prediction of kidney-related outcomes in patients with asymptomatic HFpEF [14]. Conventionally used natriuretic peptides (NPs) exhibited their inadequate discriminative potency on clinical outcomes in individuals with metabolic risk factors including those who were overweight and obese as well as those with T2DM [15]. Moreover, a relative deficiency of brain NP (BNP) was found to be associated with any phenotype of HF including those who had asymptomatic diastolic dysfunction [16]. In this connection, the discovery of novel biomarkers appears to be intriguing and promising.

Irisin is a multifunctional myokine, which is derived from skeletal muscles and cardiomyocytes due to the cleavage of fibronectin type III domain-containing 5 and contributes to mitochondrial homeostasis, fat browning, and glucose and fatty acid oxidation [17]. Therefore, irisin exerts several cardio and renal protective capabilities through regulating vascular tone, blood pressure, and mitochondrial thermogenesis and integrity as well as enhancing the over-expression of angiopoietin-Tie2, interleukin (IL)-8, IL-13, transforming growth factor (TGF)-beta, and thrombopoietin [18,19]. Finally, it protects ischemic and reperfusion injury, necrosis, apoptosis, and ferroptosis, potentiates tissue reparation, reduces the inflammatory response and improves injured cell survival, and ameliorates kidney and skeletal muscle injury, osteopenia, and myopathy through the SIRT-1/Nrf2 pathway and AMP kinase / Drp-1 and ERK1/2 signaling [20,21]. Yet, irisin in an animal model decreased serum creatinine, urea, and phosphorous levels as well as prevented vascular calcification in CKD [22]. In clinical settings, irisin demonstrated its ability to be a predictor for T2DM-related complications and symptomatic HF [23,24,25]. However, the discriminative value of irisin for kidney-related outcomes in T2DM individuals with asymptomatic HFpEF/HFmrEF remains uncertain. The purpose of this study is to elucidate whether irisin is a promising predictive biomarker for kidney-related events in patients with T2DM and concomitant asymptomatic HF.

## 2. Materials and Methods

### 2.1. Patient Population and Study Design

**Figure 1:**
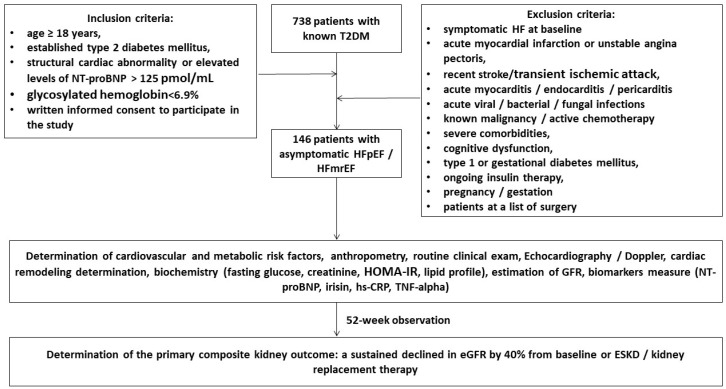
Flow chart of study design. Abbreviations: T2DM, type 2 diabetes mellitus; HFpEF, heart failure with preserved ejection fraction; HFmrEF, heart failure mildly reduced ejection fraction; NT-proBNP, N-terminal brain natriuretic pro-peptide; hs-CRP, high-sensitive C-reactive peptide; GFR, glomerular filtration rate; TNF, tumor necrosis factor.

A total of 738 patients with T2DM were identified through a local database of the “Vita Center” (Zaporozhye, Ukraine). The inclusion criteria were both genders with an age of ≥18 years, established T2DM, asymptomatic HFpEF/HFmrEF (left ventricular ejection fraction [LVEF] > 40%), glycosylated hemoglobin [HbAc1] < 6.9%, and informed consent to participate in the study. We prospectively enrolled 146 T2DM patients who had either evidence of structural cardiac abnormality or elevated levels of N-terminal brain natriuretic pro-peptide (NT-proBNP) > 125 pmol/mL and followed them for 52 weeks (Figure 1). Structural cardiac abnormalities were used as the minimum from the following criteria: abnormal left ventricular (LV) global longitudinal strain (GLS) < −16%, increased LV mass index (LVMI) ≥ 95 g/m^2^ in women or ≥115 g/m^2^ in men, left atrial volume index (LAVI) > 34 mL/m^2^, abnormal ratio of early transmitral diastolic filling velocity/early mitral annular velocity (E/e′) ≥ 13 units. These criteria corresponded to the diagnostic algorithm according to the 2021 ESC HF guidelines [26].

The exclusion criteria were symptomatic acute or chronic HF, acute coronary syndrome/myocardial infarction or unstable angina pectoris, recent stroke/transient ischemic attack, acute myocarditis/endocarditis/pericarditis, known malignancy and/or chemotherapy, acute viral/bacterial/fungal infections, severe comorbidities (anemia, chronic obstructive pulmonary disease, bronchial asthma, liver cirrhosis, known inherited and acquired valvular heart defect, symptomatic severe hypoglycemia, morbid obesity, systemic connective tissue diseases, end-stage of renal disease, autoimmune disease, cognitive dysfunction, and thyroid disorders), pregnancy/gestation, type 1 or gestational diabetes mellitus, or current therapy with insulin.

During the 52-week study period, we collected patient data from a variety of sources, including medical records, databases, discharge summaries, autopsy reports, and direct calls to patients and/or their relatives.

### 2.2. Collection of Relevant Medical Data and Background Information

Demographics and anthropomorphic data, basic clinical characteristics, and comorbidities were collected at the baseline and at the study end.

### 2.3. Echocardiography Examination

All the patients underwent echocardiographic and Doppler examinations by two blinded, highly experienced echocardiographers according to the guidelines of the American Society of Echocardiography [27]. Records were taken in the standard apical 2- and 4-chamber views at baseline and at the 52-week interval of observation using a GE HealthCare Vivid E95 scanner (General Electric Company, Horton, Norway). The conventional hemodynamic parameters included LVEF by Simpson’s method, left ventricular end-diastolic (LVEDV) and end-systolic (LVESV) volumes, LAVI, early diastolic blood filling (E), and the mean longitudinal strain ratio (e′). The estimated E/e′ ratio was expressed as the ratio of the E wave velocity to the averaged medial and lateral e’ velocity. LV GLS was received by 2D Speckle Tracking Image after obtaining high-quality echocardiographic records during at least three cardiac cycles. The data were stored using the DICOM format for further evaluation.

### 2.4. Determination of Composite Kidney Outcomes

We determined the kidney-related outcome as a composite of the estimated GFR (eGFR) reduction by 40% from baseline, ESKD, or kidney replacement therapy.

### 2.5. Blood Sampling

Venous blood samples (3–5 mL) were collected from fasting patients in vacutainer tubes at three time points: baseline, 26 weeks, and the end of the study. Pooled samples were centrifuged (3000 r/min, 30 min). Sera were collected and immediately frozen and stored at −70 °C until analysis before utilization. All the routine biochemical tests were performed using standard biochemical techniques on a Roche P800 analyzer (Basel, Switzerland).

### 2.6. Biomarker Analysis

N-terminal brain natriuretic pro-peptide (NT-proBNP), irisin, tumor necrosis factor (TNF)-alpha, and high-sensitive C-reactive protein (hs-CRP) were quantified in the serum using commercial enzyme-linked immunosorbent assay (ELISA) kits manufactured by Elabscience (Houston, TX, USA). The intra- and inter-assay coefficients of variation for all the kits were <10%.

### 2.7. Glomerular Filtration Rate and Insulin Resistance Determination

The conventional CKD-EPI formula was used to estimate the glomerular filtration rate (eGFR) [28]. The Homeostatic Assessment Model of Insulin Resistance (HOMA-IR) was used to assess insulin resistance [29].

### 2.8. Statistics

The Kolmogorov–Smirnov test was used to assess normality, and the Levene test was used to assess homogeneity. The continuous variables were presented as the mean (M) and standard deviation (SD) or median (Me) and 25–75% interquartile range (IQR), depending on their distribution. The categorical variables were presented as proportions and percentages of the total. Chi-square, Mann–Whitney U, and Kruskal–Wallis tests were used to compare variance according to the distribution. Spearman’s correlation coefficient was calculated to determine the correlation between the variables. Plausible predictors of the composite renal outcome were identified using univariate logistic regression and backward stepwise multivariate logistic regression. An odds ratio (OR) and 95% confidence interval (CI) were calculated for each predictor. The reliability of the predictive models was determined by receiver operating curve (ROC) analysis with further calculation of the area under the curve (AUC), its CI, sensitivity (Se), specificity (Sp), and likelihood ratio (LR) for each predictor. The Youden test was used to estimate the cut-off points for irisin and its trajectory. We compared the incremental prognostic capacity of the models on a binary prediction methodology based on the estimation of integrated discrimination indices (IDI) and net reclassification improvement (NRI). A two-sided *p* < 0.05 was considered significant. The variables were tested using SPSS v. 23 (IBM, Armonk, New York, NY, USA) and GraphPad Prism v. 9 (GraphPad Software, San Diego, CA, USA).

## 3. Results

### 3.1. General Characteristics of the Patients

The study participants had a mean age of 56 years and 51.7% were male (Table 1). They had the following comorbidity and CV risk profile that included dyslipidemia (78.8%), hypertension (74.0%), stable coronary artery disease (31.5%), atrial fibrillation (17.1%), abdominal obesity (37.0%), left ventricular hypertrophy (90.4%), CKD 1–3 grades (26.0%), and smoking (41.7%). All the patients had stable hemodynamics at baseline, with a mean LVEF of 47% (ranging from 41% to 51%), an LV GLS of −14.6 (−12.6; −15.9), an E/e′ of 16 ± 5 units, and moderate LV and left atrial cavity dilation. The mean LV myocardial mass index and LAVI were 138 ± 11 g/m^2^ and 41 (35–50) mL/m^2^, respectively. The mean eGFR was 78 ± 18 mL/min/1.73 m^2^, and the mean HOMA-IR was 7.10 ± 2.5 units. The mean level of NT-proBNP was 615 (168–1145) pmol/mL. All the individuals received optimal therapy depending on their clinical state, phenotype of HF, fasting glucose, lipid profile and comorbidities, which included antihypertensive agents (angiotensin-converting enzyme inhibitors [ACEi], angiotensin-II receptor blockers [ARBs], calcium channel blockers, and thiazide-like diuretics), beta-blocker and ivabradine when needed, antiplatelet agents, and statins. Patients with atrial fibrillation were treated with anticoagulants. The therapy of HFpEF/HFmrEF included beta-blockers, ACEi or ARBs, sodium-glucose cotransporter-2 (SGLT2), and thiazide-like diuretics. The therapy of T2DM included diet, metformin, SGLT2 inhibitors, DPP-4 inhibitors, and glucagon-like peptide-1 receptor agonists (GLP-1-RAs).

We found that 38 patients met the criteria for kidney-related outcomes, whereas 108 individuals did not. According to these findings, we divided the entire group into two groups. The patients from both groups did not demonstrate significant differences in gender, demographic parameters (body mass index, waist circumference, waist-to-hip ratio), CV and comorbidity profile, systolic and diastolic blood pressure, hemodynamics, E/e′, LVMMI, LAVI, HOMA-IR, fasting glucose, glycated hemoglobin, lipids, hs-CRP, TNF-alpha, and NT-proBNP. However, the patients from the kidney-related event group were older, frequently had CAD, exhibited a higher serum creatinine and urinary albumin/creatinine ratio, and lower GLS, eGFR, and irisin levels, as well as being frequently treated with beta-blockers and rarely SGLT-2 inhibitors compared with those from another group.

### 3.2. Dynamic Changes of Irisin in the Patients Included in the Study

**Figure 2:**
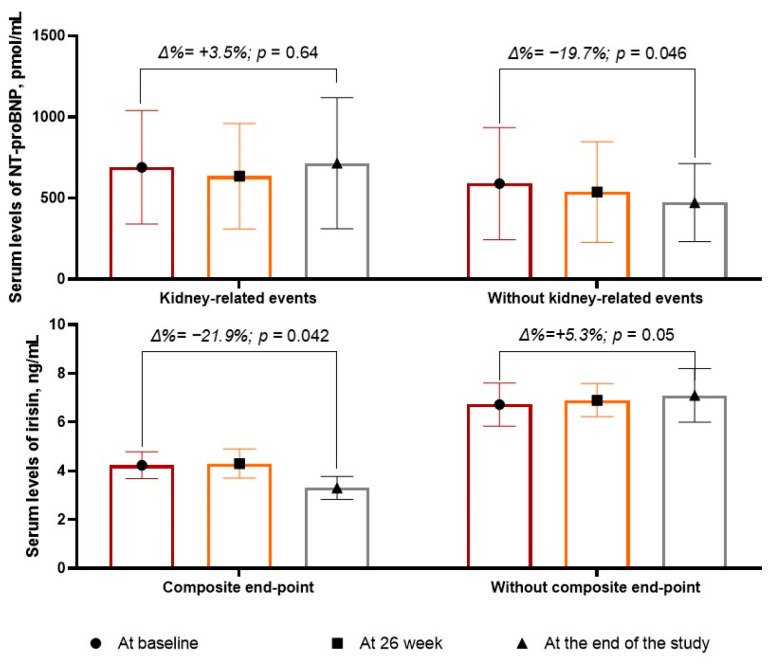
The dynamic of serum levels of NT-proBNP and irisin.

The changes in the biomarker levels during the observation period are reported in Figure 2. We found that the levels of NT-proBNP were not significantly changed in the group with the kidney-related events (Δ% = 3.5%; from 690 pmol/mL [25–75% IQR = 240–1270 pmol/mL] to 715 pmol/mL [25–75% IQR = 296–1112 pmol/mL], *p* = 0.64), whereas in the patients without kidney-related events, there was a borderline decrease in the serum levels of peptide (Δ% =−19.7%; from 589 pmol/mL [25–75% IQR = 190–994 pmol/mL] to 473 pmol/mL [25–75% IQR = 216–789 pmol/mL], *p* = 0.046).

The levels of irisin significantly (*p* = 0.05) increased in the patients without kidney-related events (Δ% = +5.3%, from 6.72 ng/mL [25–75% IQR = 5.17–8.10 ng/mL] to 7.10 ng/mL [25–75% IQR = 5.90–8.50 ng/mL], *p* = 0.05), whereas in those with kidney-related events, the levels of the biomarker decreased up to 21.9% (from 4.23 ng/mL; 25–75% IQR = 3.22–5.98 ng/mL to 3.30 ng/mL [25–75% IQR = 2.10–4.56 ng/mL]) with statistical significance (*p* = 0.042).

### 3.3. Spearman’s Correlation between the Levels of Biomarkers at Baseline and Other Parameters

The NT-proBNP levels were positively associated with E/e′ (r = 0.34, *p* = 0.001), LAVI (r = 0.31, *p* = 0.001), LV hypertrophy (r = 0.27, *p* = 0.044), and age (r = 0.22, *p* = 0.048) and were inversely associated with GLS (r = −0.35, *p* = 0.001) and eGFR (r = −0.32, *p* = 0.001). The irisin levels correlated positively with GLS (r = 0.38, *p* = 0.001) and negatively with LAVI (r = −0.32, *p* = 0.001), fasting plasma glucose (r = −0.30, *p* = 0.001), HOMA-IR (r = −0.27, *p* = 0.042), and HbA1c (r = −0.23, *p* = 0.046). There were no significant associations between the levels of circulating biomarkers, such as NT-proBNP, irisin, TNF-alpha, and hs-CRP with UACR.

### 3.4. Predictive Values of Serum Irisin at Baseline and the Trajectory of Irisin: The Results of the ROC Curve Analysis

**Figure 3:**
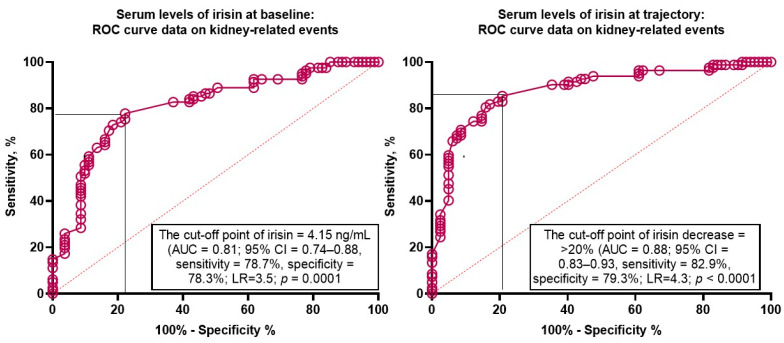
Receiver operator curve analysis for kidney-related events: The optimal cut-off points of irisin levels at baseline and the trajectory to decrease. Abbreviations: AUC, area under the curve; CI, confidence interval; LR, likelihood ratio.

We found that serum levels of irisin < 4.15 ng/mL (area under the curve [AUC] = 0.81; 95% confidence interval [CI] = 0.74–0.88; sensitivity [Se] = 78.7%, specificity [Sp] = 78.3%; likelihood ratio [LR] = 3.5; *p* = 0.0001) predicted kidney-related outcomes (Figure 3). Yet, a trend to decrease the levels of irisin (>20% from the baseline) exerted higher discriminative potency (AUC = 0.88; 95% CI = 0.83–0.93; Se = 82.9%, Sp = 79.3%; LR = 4.3; *p* < 0.0001) than its baseline level.

### 3.5. Predictors for Kidney-Related Events in T2DM Patients with Asymptomatic HFpEF: Univariate and Multivariate Logistic Regression Analysis

We used the median value for age and NT-proBNP at baseline for this analysis (Table 2). The univariate logistic regression unveiled that kidney-related events were not predicted by an age > 61 years (odds ratio [OR] = 1.03; *p* = 0.464) and serum levels of NT-proBNP ≥ 690 pmol/mL (OR = 1.06; *p* = 0.834), whereas irisin at a baseline of ≤ 4.15 ng/mL (OR = 1.16; *p* = 0.001), a decrease in irisin of >20% (OR = 1.24; *p* = 0.001), UACR (OR = 1.07; *p* = 0.012), and the use of SGLT2i (OR = 0.91; *p* = 0.044) did predict kidney-related events.

The multivariate logistic regression revealed that irisin at baseline ≤ 4.15 ng/mL (OR = 1.14; *p* = 0.016), a decrease in irisin > 20% (OR = 1.27; *p* = 0.001), and the use of SGLT2i (OP = 0.94; *p* = 0.041) were independently associated with kidney-related events. The UACR exerted borderline significance for this dependent variable (OP = 1.04; *p* = 0.050).

### 3.6. Comparison of the Predictive Models

We compared predictive models for kidney-related outcomes and found that Model 1 (the discriminative value of irisin < 4.15 ng/mL) was not superior to Model 3 (administration of SGLT2i), whereas Model 2 (decrease in irisin levels >20%) was significantly better than the reference value (Table 3). Model 1 + Model 2 demonstrated better discriminative potency in comparison with Model 1, whereas Model 3 did not add sufficient predictive information to Model 2.

### 3.7. Reproducibility of Biomarker Trajectory

The evaluation of the reproducibility of the irisin trajectory was performed in comparison with NT-proBNP. The intra-class correlation coefficient for the inter-observer reproducibility of NT-proBNP was 0.94 (95% CI = 0.89–0.97), whereas the intra-class correlation coefficient for the intra-observer reproducibility of irisin was 0.92 (95% CI = 0.88–0.95).

## 4. Discussion

The results of the study revealed that levels of irisin ≤ 4.15 ng/mL and its decrease > 20% from the baseline level showed independent discriminative potency for kidney-related events in T2DM patients with asymptomatic HF. Recently, we established that irisin was a better predictor for the renoprotective effect of the SGLT2 inhibitor dapagliflosin in diabetics with chronic symptomatic HF, regardless of its phenotypes [30]. However, the predictive value of irisin at baseline and its trajectory for long-term observation among T2DM with concomitant asymptomatic HFpEF/HFmrEF was found first.

Although irisin has been previously considered a multifunctional myokine, which is activated by peroxisome proliferator-activated receptor γ coactivator-1α (PGC-1α) during physical exercise in skeletal muscle tissues and thereby links metabolic homeostasis with cardioprotection, its renoprotective capability remains uncertain [31,32]. However, low levels of irisin were found in several metabolic conditions (obesity, metabolic syndrome, metabolic fatty liver syndrome, peri-/post-menopausal osteoporosis) and cardiovascular diseases including heart failure, acute myocardial infarction, and stable coronary artery disease [33,34,35,36]. Nevertheless, lower irisin concentrations were found to be a powerful predictor for poor clinical outcomes, including premature death, in acute myocardial infarction and any phenotype of HF [37,38,39]. At the same time, an increase in irisin plasma levels in HFpEF/HFrEF individuals was associated with an improved clinical status, functional and structural cardiac performances, anti-oxidative, anti-fibrotic and anti-inflammatory effects, which was translated into a reduction in adverse outcomes [30,40,41].

It has been suggested that irisin is able to inhibit angiotensin-II-induced fibroblast trans-differentiation, collagen production, inflammation, and oxidative stress through the suppression of LOXL2 and TGF-β1 as well as via the phosphorylation of Smad2/3 and SIRT-related molecular mechanisms [41,42]. Indeed, several studies of HF, including HF-induced cachexia, have shown a negative correlation between the irisin concentration and hs-CRP, TNF-alpha, and interleukin-6 [43,44,45]. We did not find a sufficient association between irisin concentrations and inflammatory cytokines, such as hs-CRP and TNA-alpha, in patients without clinical presentation of cardiac dysfunction, while the levels of irisin were found to be decreased. Yet, among patients with asymptomatic HFpEF/HFmrEF, there were no serious differences in hemodynamics besides LV GLS depending on the presence of kidney-related events, whereas irisin concentrations were significantly lower in the group with kidney-related events compared with those who did not have a kidney-related event. These findings seem to show that the regulation of irisin and underlying molecular mechanisms, which correspond to the remodeling of remote organs, could distinguish patients with acute HF, severe HFrEF, or cardiac cachexia.

Cumulatively, we suggested that kidney protective mechanisms in T2DM with a conventional comorbidity profile (overweight, mild-to-moderate obesity, dyslipidemia, mild fasting hyperglycemia) are likely to be mediated by irisin, which is not only produced by skeletal muscles but also white adipose tissue and perivascular/pericardial adipose tissues and also acts through both local and central nervous system-related mechanisms, such as enhancing organ perfusion, inducing protective autophagy, alleviating apoptosis and fibrosis, improving kidney function, regulating incretin metabolism, and adipose tissue browning [44,45]. Consequently, irisin is involved in both cardiac and kidney protection by interacting with crucial signaling pathways in regulating glucose metabolism, lipid homeostasis, fibrosis, and inflammation [46]. As a result, a decreasing concentration of irisin is considered a key factor in the inadequate regulation of the endogenous repair system. Indeed, in animal studies, reduced skeletal muscle FNDC5 expression in ischemic cardiomyopathy was likely modulated by inflammatory cytokines and/or angiotensin-II via the down-regulated PGC-1α, which corresponded to low irisin production [47,48].

The lack of a significant predictive value of NT-proBNP in T2DM patients with asymptomatic cardiac dysfunction is another issue that requires a detailed explanation. It appeared that in this group of patients, the peptide did not demonstrate a diagnostically significant trend to predict renal events. Overall, we established that either low levels of irisin or a decrease in its concentration were more powerful predictors for the worsening of kidney function and related outcomes than NPs. Although the inclusion criteria were designed to avoid the overrepresentation of patients with low peptide values, these data confirm the limited ability of NPs to predict renal events in patients with HF in the absence of clinical manifestations and volume overload. Indeed, recent clinical trials for SGLT2i have shown that the benefits of these agents may be extended to patients regardless of T2DM on clinically meaningful HF [49,50]. At the same time, it remains unclear whether measures of NT-proBNP appear to be effective in predicting HF and non-HF outcomes in SGLT2i-treated individuals. The results of the study suggest that low levels of irisin deserve to be serially measured as a plausible predictive biomarker of a kidney-related clinical outcome. Of note, irisin also possesses anti-inflammatory, anti-oxidative, and anti-apoptotic properties, which make it associated with a transition from acute kidney injury to CKD as well as the development of CKD [51].

Although the patients who were included in the study had other reasons for reduced irisin levels besides T2DM, such as CAD, being obese, or being overweight, this does not explain why the lowest myokine concentration was found specifically in the group of patients who had kidney-related events. The comorbidities found are common in both diabetic patients and those with asymptomatic HF. Interestingly, SGLT2i treatment prevented, to some extent, the decrease in circulating irisin levels, which probably explains the obtained correlation between the administration of these drugs and the reduced risk of renal events. The positive effects of SGLT2i on renal structure and function as well as clinically relevant renal events are considered established in many randomized clinical trials [52,53]. However, the underlying mechanism of action by which this class of drugs can improve patient survival and prevent progressive deterioration of renal function remains a matter of scientific debate. The results of our study open prospects for understanding the pathophysiological role of irisin in the implementation of renoprotection and may have an impact on improving approaches to predict adverse renal events in the absence of other potential factors. At the same time, the mechanism by which the renoprotective effect of irisin is translated in T2DM patients with asymptomatic HFpEF/HFmrEF remains unclear and requires further research.

### Study Limitations

The study has several limitations. First, we did not evaluate the metabolic and nutritional status of the patients; however, we recommended enhancing a convenient nutrition plan for them during the period of observation. Second, all the T2DM individuals were well treated with a combination of diet and contemporary agents to reach optimal glycaemia control. In fact, the results of the study seem to be extrapolated to a similar population. Third, we did not investigate in the study the role of SGLT2i, which was used as an antidiabetic agent. In addition, in our analyses, we did not evaluate the level of physical activity. Finally, we believe that these limitations will not intervene in the interpretation of the findings.

## 5. Conclusions

We established that a low baseline level of irisin (≤4.15 ng/mL) and its 20% decrease seem to be independently associated with newly kidney-related events in T2DM patients with asymptomatic cardiac dysfunction. The findings are likely to gain the perspectives of the personifying treatments of asymptomatic HFpEF/HFmrEF in T2DM to prevent kidney-related outcomes.

## Figures and Tables

**Table 1:** General characteristics of eligible patients at admission.

Variables	Entire Group (*n* = 146)	Kidney-Related Event Group (*n* = 38)	Kidney-Related Event Free Group (*n* = 108)	*p* Value
Age, years	56 (46–66)	61 (52–68)	54 (44–67)	0.048
Male/female *n* (%)	84 (51.7)/62 (49.3)	21 (55.2)/17 (44.8)	63 (58.3)/45 (41.6)	0.758
BMI, kg/m^2^	25.8 ± 2.90	26.1 ± 2.60	25.2 ± 2.80	0.680
Waist circumference, cm	96.6 ± 3.70	99.4 ± 3.80	94.7 ± 3.77	0.660
WHR, units	0.88 ± 0.07	0.90 ± 0.09	0.85 ± 0.06	0.840
Dyslipidemia, *n* (%)	115 (78.8)	30 (78.9)	85 (78.7)	0.744
Hypertension, *n* (%)	108 (74.0)	29 (76.3)	79 (73.1)	0.680
Stable CAD, *n* (%)	46 (31.5)	15 (39.5)	31 (28.7)	0.042
Atrial fibrillation, *n* (%)	25 (17.1)	7 (18.4)	18 (16.7)	0.261
Smoking, *n* (%)	61 (41.7)	17 (44.7)	44 (40.7)	0.652
Abdominal obesity, *n* (%)	54 (37.0)	14 (36.8)	40 (37.0)	0.840
LVH *, *n* (%)	132 (90.4)	35 (92.1)	97 (89.8)	0.050
CKD 1–3 grades, *n* (%)	38 (26.0)	11 (28.9)	27 (25.0)	0.064
Systolic BP, mm Hg	138 ± 9	140 ± 8	137 ± 7	0.612
Diastolic BP, mm Hg	82 ± 8	87 ± 6	79 ± 9	0.712
LVEDV, mL	154 (134–163)	156 (139–166)	153 (132–163)	0.434
LVESV, mL	82 (61–93)	86 (82–103)	81 (67–97)	0.667
LVEF, %	47 (41–51)	45 (41–49)	47 (42–52)	0.854
LVMMI, g/m^2^	138 ± 11	146 ± 15	133 ± 13	0.162
LAVI, mL/m^2^	41 (35–50)	42 (38–52)	41 (35–49)	0.064
E/e′, unit	16 ± 5	17 ± 4	16 ± 4	0.448
GLS, %	−14.6 (−12.6; −15.9)	−12.4 (−10.2; −14.3)	−14.3 (−11.4; −15.8)	0.024
eGFR, mL/min/1.73 m^2^	78 ± 18	66 ± 12	87 ± 12	0.045
UACR (mg/g Cr)	19.8 (5.6–32.9)	23.3 (6.3–34.1)	14.1 (4.9–23.9)	0.046
HOMA-IR, units	7.10 ± 2.5	7.36 ± 2.9	7.05± 2.3	0.721
Fasting glucose, mmol/L	6.45 ± 1.8	6.58 ± 1.5	6.31 ± 1.7	0.882
HbA1c, %	6.42 ± 0.16	6.61 ± 0.14	6.21 ± 0.15	0.445
Creatinine, µmol/L	101.2 ± 19.8	146.6 ± 34.3	98.7 ± 28.5	0.040
SUA, mcmol/L	318 ± 112	387 ± 105	306 ± 88	0.052
Total cholesterol, mmol/L	5.98 ± 1.22	6.03 ± 1.33	5.90 ± 1.18	0.546
HDL-C, mmol/L	0.97 ± 0.21	0.95 ± 0.23	0.99 ± 0.20	0.548
LDL-C, mmol/L	3.30± 0.25	3.32± 0.25	3.21 ± 0.22	0.460
Triglycerides, mmol/L	1.38 ± 0.23	1.41 ± 0.24	1.43 ± 0.19	0.424
hs-CRP, mg/L	4.32 (2.43–6.80)	4.56 (2.65–7.10)	4.15 (2.17–6.32)	0.070
TNF-alpha, pg/mL	2.66 (1.80–3.70)	2.92 (1.85–3.80)	2.54 (1.75–3.61)	0.726
NT-proBNP, pmol/mL	615 (168–1145)	690 (240–1270)	589 (190–994)	0.480
Irisin, ng/mL	5.45 (3.30–7.75)	4.23 (3.22–5.98)	6.72 (5.17–8.10)	0.012
ACE inhibitors, *n* (%)	56 (38.4)	13 (34.2)	43 (39.8)	0.672
Angiotensin-II receptor blockers, *n* (%)	52 (35.6)	16 (42.1)	36 (33.3)	0.054
Beta-blockers, *n* (%)	46 (31.5)	15 (39.5)	31 (28.7)	0.042
Ivabradine, *n* (%)	32 (21.9)	9 (23.7)	23 (21.3)	0.722
Calcium channel blockers, *n* (%)	41 (28.1)	11 (28.9)	30 (27.8)	0.728
Thiazide-like diuretics, *n* (%)	43 (29.4)	10 (26.3)	33 (30.6)	0.464
Antiplatelet, *n* (%)	121 (81.8)	32 (84.2)	89 (82.4)	0.880
Anticoagulants, *n* (%)	25 (17.1)	7 (18.4)	18 (16.7)	0.261
Metformin, *n* (%)	146 (100.0)	38 (100.0)	108 (100.0)	1.000
DPP4 inhibitor, *n* (%)	21 (14.4)	5 (13.3)	16 (14.8)	0.846
GLP-1 receptor agonist, *n* (%)	26 (17.8)	7 (18.4)	19 (17.6)	0.723
SGLT2 inhibitors, *n* (%)	119 (81.5)	29 (76.3)	90 (83.3)	0.048
Statins, *n* (%)	133 (91.1)	33 (86.8)	100 (92.6)	0.070

Notes: Variables are given as M ± SD and Me (25–75% IQR). The chi-square test was used to compare categorical variables. The Mann–Whitney U test and Kruskal–Wallis test were used to compare continuous variables between cohorts. * LVH was detected when LVMI ≥95 g/m^2^ in women or ≥115 g/m^2^ in men. Abbreviations: BMI, body mass index; CAD, coronary artery disease; CKD, chronic kidney disease; eGFR, estimated glomerular filtration rate; E/e′, early diastolic blood filling to longitudinal strain ratio; GLS, global longitudinal strain; GLP-1, glucagon-like peptide-1; HbA1c, glycated hemoglobin; HDL-C, high-density lipoprotein cholesterol; HOMA-IR, Homeostatic Assessment Model of Insulin Resistance; hs-CRP, high-sensitivity C-reactive protein; LAVI, left atrial volume index; LDL-C, low-density lipoprotein cholesterol; LVH, left ventricular hypertrophy; LVEDV, left ventricular end-diastolic volume; LVESV, left ventricular end-systolic volume; LVEF, left ventricular ejection fraction; LVMMI, left ventricle myocardial mass index; NT-proBNP, N-terminal natriuretic pro-peptide; SGLT2, sodium-glucose cotransporter-2; SUA, serum uric acid; TNF-alpha, tumor necrosis factor-alpha; UACR, urinary albumin/creatinine ratio; WHR, waist-to-hip ratio.

**Table 2:** Predictors for kidney-related events: The results of the logistic regression.

Variables	Dependent Variable: Kidney-Related Events					
	Univariate Log Regression			Multivariate Log Regression		
	OR	95% CI	*p* Value	OR	95% CI	*p* Value
Age ≥61 vs. <61 years	1.03	1.00–1.06	0.464	-		
NT-proBNP at baseline ≥690 pmol/mL vs. < 690 pmol/mL	1.06	0.93–1.88	0.834	-		
Irisin at baseline ≤ 4.15 ng/mL vs. >4.15 ng/mL	1.16	1.07–2.40	0.001	1.14	1.02–2.10	0.016
Decrease in irisin levels >20% vs. ≤20%	1.24	1.12–2.56	0.001	1.27	1.10–2.55	0.001
UACR	1.07	1.02–1.13	0.012	1.04	1.01–1.09	0.050
Administration of SGLT2i vs. unused SGLT2i	0.91	0.86–0.98	0.044	0.94	0.88–0.96	0.041

Abbreviations: OR, odds ratio; CI, confidence interval; SGLT2i, sodium-glucose cotransporter-2 inhibitor; UACR, urinary albumin/creatinine ratio; NT-proBNP, N-terminal natriuretic pro-peptide.

**Table 3:** The comparisons of predictive models for kidney-related outcomes.

Predictive Models	Dependent Variable: Kidney-Related Events					
	AUC		NRI		IDI	
	M (95% CI)	*p* Value	M (95% CI)	*p* Value	M (95% CI)	*p* Value
Model 1 (Irisin at baseline ≤ 4.15 ng/mL)	0.811 (0.773–0.857)	-	Reference	-	Reference	-
Model 2 (Decrease in irisin levels > 20%)	0.893 (0.834–0.951)	0.001	0.61 (0.57–0.67)	0.022	0.55 (0.50–0.61)	0.020
Model 3 (Administration of SGLT2i)	0.813 (0.791–0.836)	0.644	0.18 (0.15–0.23)	0.224	0.19 (0.15–0.25)	0.142
Model 1 + Model 2	0.922 (0.855–0.973)	0.050	0.32 (0.26–0.38)	0.020	0.34 (0.30–0.39)	0.022
Model 2 + Model 3	0.899 (0.861–0.963)	0.264	0.14 (0.11–0.19)	0.660	0.25 (0.19–0.32)	0.548

Abbreviations: AUC, area under curve; CI, confidence interval; IDI, integrated discrimination indices; NRI, net reclassification improvement. Note: *p* value indicates a significant difference in terms of Model 1.

## Data Availability

The data presented in this study are available on request from the corresponding author due to privacy restrictions.
